# Clinicopathological Spectrum of Brain Parenchymal Infections: A Retrospective Analytical Study

**DOI:** 10.7759/cureus.110777

**Published:** 2026-06-13

**Authors:** Rahul G Tiwari, Archana Balasubramanian, Leena Dennis Joseph, Lawrence D'Cruze, Rajeswaran R, Visvanathan Krishnaswamy, Bhaskar Naidu P

**Affiliations:** 1 Department of Pathology, Sri Ramachandra Institute of Higher Education and Research, Chennai, IND; 2 Department of Diagnostic Radiology, Sri Ramachandra Institute of Higher Education and Research, Chennai, IND; 3 Department of Neurosurgery, Sri Ramachandra Institute of Higher Education and Research, Chennai, IND

**Keywords:** central nervous system infections, clinicopathological correlation, cryptococcosis, granulomatous inflammation, histopathology, mucormycosis, neurotuberculosis, spinal infections

## Abstract

Introduction: Brain parenchymal infections encompass a broad clinicopathological spectrum caused by bacteria, mycobacteria, fungi, and parasites, presenting non-specifically with headache, fever, seizures, focal neurological deficits, and altered sensorium. *Mycobacterium tuberculosis* remains the most prevalent causative organism in India, while opportunistic pathogens, including *Cryptococcus*, *Mucor*, and *Zygomycetes *species, are increasingly encountered in immunocompromised hosts. Although neuroimaging and microbiological investigations are integral to evaluation, imaging features frequently overlap with inflammatory and neoplastic processes, and microbiological methods are limited by suboptimal sensitivity. Histopathological examination remains indispensable for definitive diagnosis, characterising the inflammatory response and guiding therapeutic management. This study evaluates the clinicopathological spectrum of neuroparenchymal infections, excluding meningitis, at a single tertiary care centre in India.

Methods: This clinicopathological case series included 12 patients who were diagnosed with infections of the central nervous system and spine over a period of two years, from October 2023 to October 2025, at a tertiary care centre. Clinical findings, radiological diagnoses, and microbiological investigation results were collected and correlated with histopathological findings from archived paraffin-embedded blocks.

Results: The study comprised eight females (66.7%) and four males (33.3%). The age of the study population ranged from 15 to 65 years (mean ± SD: 45.6 ± 13.2 years). Histopathological examination revealed a wide spectrum of lesions: granulomatous inflammation, three cases (25.0%); necrotising granulomatous inflammation, two cases (16.7%); xanthogranulomatous inflammation, two cases (16.7%); chronic inflammatory process, two cases (16.7%); acute-on-chronic inflammation, one case (8.3%); organised bacterial abscess, one case (8.3%); cryptococcal infection, one case (8.3%); and mucormycosis/fungal abscess, two cases (16.7%). Clinicopathological concordance was observed in 9 of 12 cases (75%). In 3/12 discordant cases (25%), histopathological findings differed significantly from the clinical and radiological diagnoses. The chi-square test was used to assess the association between the clinical and radiological impression and the final histopathological diagnosis (χ² = 9.6, p < 0.05).

Conclusion: This series highlights the important role of histopathological examination in the accurate diagnosis of neuroinfections. An integrated diagnostic approach, including clinical, radiological, microbiological, and histopathological findings, is essential for favourable patient outcomes.

## Introduction

Brain parenchymal infections span a diverse spectrum of pathogens and histopathological stages. They range from acute, diffuse cerebritis to localised, walled-off abscesses and chronic granulomas. Accurate diagnosis relies on integrating clinical presentation, neuroimaging, and histopathology to identify the underlying microbiological agent and prevent significant neurological sequelae. The causative organisms include bacteria, mycobacteria, fungi, and parasites. The clinical manifestations are often non-specific, most commonly presenting as headache, fever, seizures, focal neurological deficits, and altered sensorium.

Tuberculosis of the central nervous system (CNS), caused by *Mycobacterium tuberculosis*, remains one of the most prevalent infective aetiologies in India and can manifest as meningitis, tuberculoma, spinal Pott's disease, or cerebral abscess [1-3]. Neuroinfections caused by various other pathogens, such as *Cryptococcus *species, *Mucor *species, *Entamoeba, Burkholderia*, *Staphylococcus aureus*, and *Zygomycetes *species, have also been increasingly reported [4,5]. These infections are particularly common in immunocompromised individuals, including older adults and individuals affected by diabetes mellitus or chronic kidney disease.

Neuroimaging, particularly contrast-enhanced computed tomography (CT) and magnetic resonance imaging (MRI), plays an important role in the initial evaluation of CNS lesions; however, imaging features frequently overlap among infective, inflammatory, and neoplastic processes [3,6,7]. Microbiological investigations, such as GeneXpert, acid-fast bacilli (AFB) smear, bacterial culture, serological investigations, and fungal studies, are invaluable but have limitations in sensitivity and may yield false-negative results [8].

Hence, histopathological examination (HPE) of tissue specimens remains the cornerstone of definitive diagnosis [9,10]. HPE not only confirms the presence of infection but also characterises the type of inflammatory response, such as granulomatous, necrotising, suppurative, or xanthogranulomatous, thereby aiding treatment planning. This study aims to evaluate the clinicopathological spectrum of neuroparenchymal infections, excluding meningitis, at a single tertiary care centre in India.

## Materials and methods

This retrospective observational case series was conducted in the Department of Pathology at Sri Ramachandra Medical College & Research Institute over a two-year period (October 2023 to October 2025). The study was performed in accordance with the ethical principles of the Declaration of Helsinki and was approved by the Institutional Ethics Committee (IEC Ref No: CSP-MED/26/APR/127/117). Twelve cases of CNS and spinal infections were included.

Inclusion criteria

The following cases were included: (1) patients diagnosed with infectious lesions of the brain parenchyma through histopathological examination; (2) cases in which a tissue diagnosis was obtained through biopsy or surgical excision; and (3) cases with available clinical and radiological findings.

Exclusion criteria

The following cases were excluded: (1) CNS infections that were not parenchymal (e.g., meningitis); (2) tissue samples that were inadequate or inadequately preserved; and (3) cases with absent or incomplete clinical and/or radiological data.

Data collection

Demographic data, presenting complaints, laboratory investigations, microbiological findings, radiological diagnosis, and final histopathological diagnosis were collected from the laboratory and hospital information systems.

Statistical analysis

Data were analysed using descriptive statistics. Continuous variables are expressed as mean and standard deviation. Categorical variables are reported as absolute numbers (N) and percentages (%). Clinicopathological concordance was assessed by comparing the final HPE diagnosis with the initial clinical and radiological diagnosis for each case. The chi-square (χ²) test was used to evaluate the association between radiological diagnosis and final HPE diagnosis, with statistical significance set at p < 0.05.

Microbiological investigations, including GeneXpert for acid-fast bacilli, AFB smear by Ziehl-Neelsen staining, aerobic bacterial culture, antibiotic sensitivity testing, Gram stain, and fungal studies, including potassium hydroxide (KOH) preparation and fungal culture on Sabouraud dextrose agar, were collected.

Assessment of radiographic images

Radiological findings were extracted from hospital records and radiology reports. Each patient underwent computed tomography (CT), magnetic resonance imaging (MRI), or both, in accordance with the clinical indication.

The imaging findings were classified as infectious, neoplastic, or indeterminate for the purpose of radiological interpretation. Methodical acquisition and analysis of information, as well as systematic documentation of clinical and pathological findings, were performed.

## Results

Demographics

The study included 12 patients with CNS and spinal infections (Table 1). Demographic data are summarised in Table 2. Among them, eight patients (66.7%) were female and four patients (33.3%) were male. The age ranged from 15 to 65 years, with a mean age of 45.6 ± 13.2 years. Most patients (9/12, 75%) belonged to the 40-65-year age group. One adolescent patient (aged 15 years) was also included. Nine of 12 patients (75%) were immunocompetent, and 3/12 (25%) were immunocompromised.

**Table 1:** Summary of clinicopathological, microbiological, and histopathological findings (N = 12). Data are presented as individual case values. SOL: space-occupying lesion, TLC: total leucocyte count, BSL: blood sugar level, ESR: erythrocyte sedimentation rate, AFB: acid-fast bacilli, HPE: histopathological examination, CD68: cluster of differentiation 68, GBM: glioblastoma multiforme, CKD: chronic kidney disease, TB: tuberculosis.

Case No.	Age/Sex	Clinical Presentation	Radiological Findings	Microbiology	Lab Parameters	Clinico-radio Impression	HPE Diagnosis
1	53/F	Low back ache	L3-L4 degenerative spondylosis	Culture: No growth; GeneXpert: Negative	TLC 17,000/µL; Polymorphs 80%; BSL 400 mg/dL	Infective	Xanthogranulomatous inflammation
2	20/F	Headache, diplopia, vomiting	Ill-defined heterogeneous hypodense SOL, frontal lobe with oedema	GeneXpert & Culture: Negative	TLC 18,000/µL; Polymorphs elevated; Immunocompetent	Neoplastic (possible)	Granulomatous inflammation
3	30/F	Headache	CT brain: normal	GeneXpert & Culture: Negative	All parameters normal	No abnormality	Xanthogranulomatous inflammation (CD68+)
4	48/F	Headache (2 yrs), seizures (10 days)	Well-defined heterogeneous enhancing lesion, temporal lobe	GeneXpert: Positive; AFB: Positive; Fungus: Negative	BSL 228 mg/dL	Infective (TB)	Necrotizing granulomatous inflammation (TB)
5	57/M	Back pain	Extradural extramedullary lesion D2–D7	GeneXpert: Negative	ESR 122 mm/hr; TLC 15,000/µL	Infective	Chronic inflammatory process / Granulomatous inflammation
6	65/M	Headache, fever	Frontal and temporal lobe lesion	AFB: Negative; Gram stain: *Staphylococcus aureus*	Pyrexia	Infective/neoplastic	Organised bacterial abscess
7	62/M	Headache, seizures	Suggestive of metastatic adenocarcinoma	All microbiology normal	Normal	Neoplastic (metastasis)	Cryptococcal infection (fungal)
8	15/F	Limb weakness	Cerebral melioidosis/inflammatory changes	Culture: *Burkholderia pseudomallei*	Normal	Infective	Acute on chronic inflammation (Melioidosis)
9	40/F	Back pain	X-ray D5–D6: Infective degenerative changes	GeneXpert: Low detected; AFB: Positive	ESR 76 mm/hr; Immunocompetent	Infective (TB)	Necrotising granulomatous inflammation (TB)
10	55/M	Headache, diplopia	Suggestive of mucormycosis	Fungal culture: Broad aseptate hyphae (Mucor)	BSL 213 mg/dL	Infective (fungal)	Mucormycosis
11	48/M	New onset seizures	MRI: Neoplasm? GBM	All negative	Biochemically deranged; Immunocompromised	Neoplastic (GBM)	Granulomatous inflammation with AFB detected (TB)
12	54/M	Limb weakness, occipital headache (15 days)	MRI: Hyperintense parieto-occipital lesion, cerebral abscess	Fungal & TB: Negative	Immunocompromised (CKD)	Infective	Fungal abscess - Zygomycetes (Mucormycosis)

**Table 2:** Demographic characteristics of the study population (N = 12). Data are presented as N (%) for categorical variables and mean ± SD for continuous variables. Descriptive statistics only; no inferential test applied. Statistical significance threshold: p < 0.05.

Demographic Parameter	N (%)	Value
Total patients	12 (100%)	–
Sex: Female	8 (66.7%)	–
Sex: Male	4 (33.3%)	–
Age range (years)	–	15–65
Mean age (years)	–	45.6 ± 13.2
Age group: ≤18 years	1 (8.3%)	–
Age group: 19–39 years	2 (16.7%)	–
Age group: 40–65 years	9 (75%)	–
Immunocompromised	3 (25%)	–
Immunocompetent	9 (75%)	–

Clinical presentation

Clinical manifestations were variable and non-specific, as summarised in Table 3. Headache was the most common presenting symptom, seen in 7/12 cases (58.3%), followed by back pain or limb weakness in 4/12 cases (33.3%). Seizures were noted in 3/12 patients (25.0%), fever in 2/12 cases (16.7%), and diplopia in 2/12 cases (16.7%), specifically in Case 2 and Case 10, both later confirmed to have CNS infections. Underlying immunocompromised states were identified in three patients: diabetes mellitus was present in Case 11 and Case 12, with Case 12 additionally having chronic kidney disease with renal failure.

**Table 3:** Clinical presentation in study patients (N = 12). Data are presented as N (%). Frequencies were compared using the chi-square (χ²) test where applicable; results are reported as χ² (df), p-value. Statistical significance threshold: p < 0.05.

Symptom	N	%	Cases
Headache	7	58.3	1,2,3,4,6,7,11
Back pain/limb weakness	4	33.3	5,8,9,12
Seizures	3	25.0	4,7,11
Fever	2	16.7	6,8
Diplopia	2	16.7	2,10

Radiological findings

**Figure 1:**
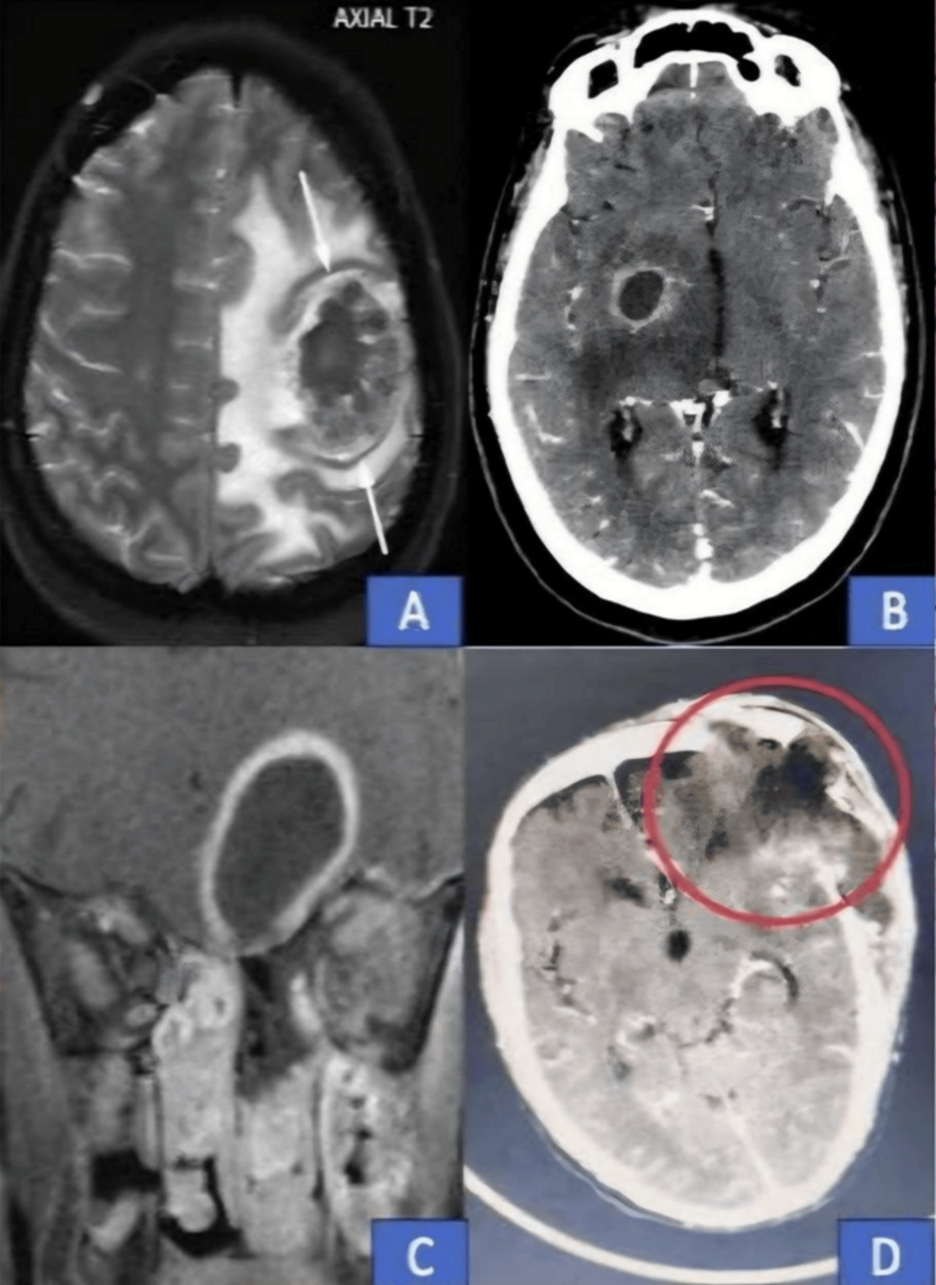
Radioimaging findings. (A) T2-weighted image showing granulomatous inflammation; (B) ring-enhancing lesions in neuromelioidosis; (C) abscess in mucormycosis; (D) infected bone with abscess. (Cases referred to in the text are Cases 4, 8, 10, and 6, respectively.)

Radiological evaluation using CT and/or MRI was available for all 12 patients, as shown in Table 4 and Figure 1. Imaging suggested infective or inflammatory pathology in 8/12 cases (66.7%). Three cases (25.0%), Case 2, Case 7, and Case 11, were radiologically interpreted as possible neoplastic lesions (suspected adenocarcinoma, glioblastoma multiforme, or lymphoma). In Case 7 (62-year-old male), imaging was suggestive of metastatic adenocarcinoma; however, HPE demonstrated cryptococcal infection. One patient (Case 3) showed no significant abnormality on imaging.

**Table 4:** Radiological findings in study patients (N = 12). Data are presented as N (%). Frequencies were compared using the chi-square (χ²) test where applicable; results are reported as χ² (df), p-value. Statistical significance threshold: p < 0.05.

Radiological Impression	N	%	Cases
Infective/inflammatory	8	66.7	1,4,5,6,8,9,10,12
Neoplastic (suspected)	3	25.0	2,7,11
No significant abnormality	1	8.3	3

Microbiological findings

Microbiological investigations were inconclusive or negative in the majority of cases (7/12, 58.3%), as summarised in Table 5. GeneXpert MTB/RIF assay was positive in 2/12 cases (16.7%), Case 4 and Case 9, both of which also demonstrated AFB positivity on ZN staining. Case 11 showed AFB positivity on histopathology despite negative microbiological studies. Gram stain and bacterial culture identified Staphylococcus aureus in Case 6 (1/12, 8.3%). Fungal cultures were positive for mucormycosis in Case 10 and for Cryptococcus species in Case 7 (1/12, 8.3% each). Burkholderia pseudomallei was isolated in Case 8 (1/12, 8.3%).

**Table 5:** Microbiological findings in study patients (N = 12). Data are presented as N (%). Frequencies were compared using the chi-square (χ²) test where applicable; results are reported as χ² (df), p-value. Statistical significance threshold: p < 0.05. MTB/RIF: Mycobacterium tuberculosis/rifampicin, AFB: acid-fast bacilli, df: degrees of freedom.

Microbiological Finding	N	%	Cases
Inconclusive/negative	7	58.3	1,2,3,5,7,11,12
GeneXpert MTB/RIF positive	2	16.7	4,9
AFB smear positive	3	25.0	4,9,11
Bacterial culture (*S. aureus*)	1	8.3	6
Fungal culture (Mucormycosis)	1	8.3	10
Fungal culture (Cryptococcus)	1	8.3	7
Burkholderia pseudomallei	1	8.3	8

Histopathological findings

**Figure 2:**
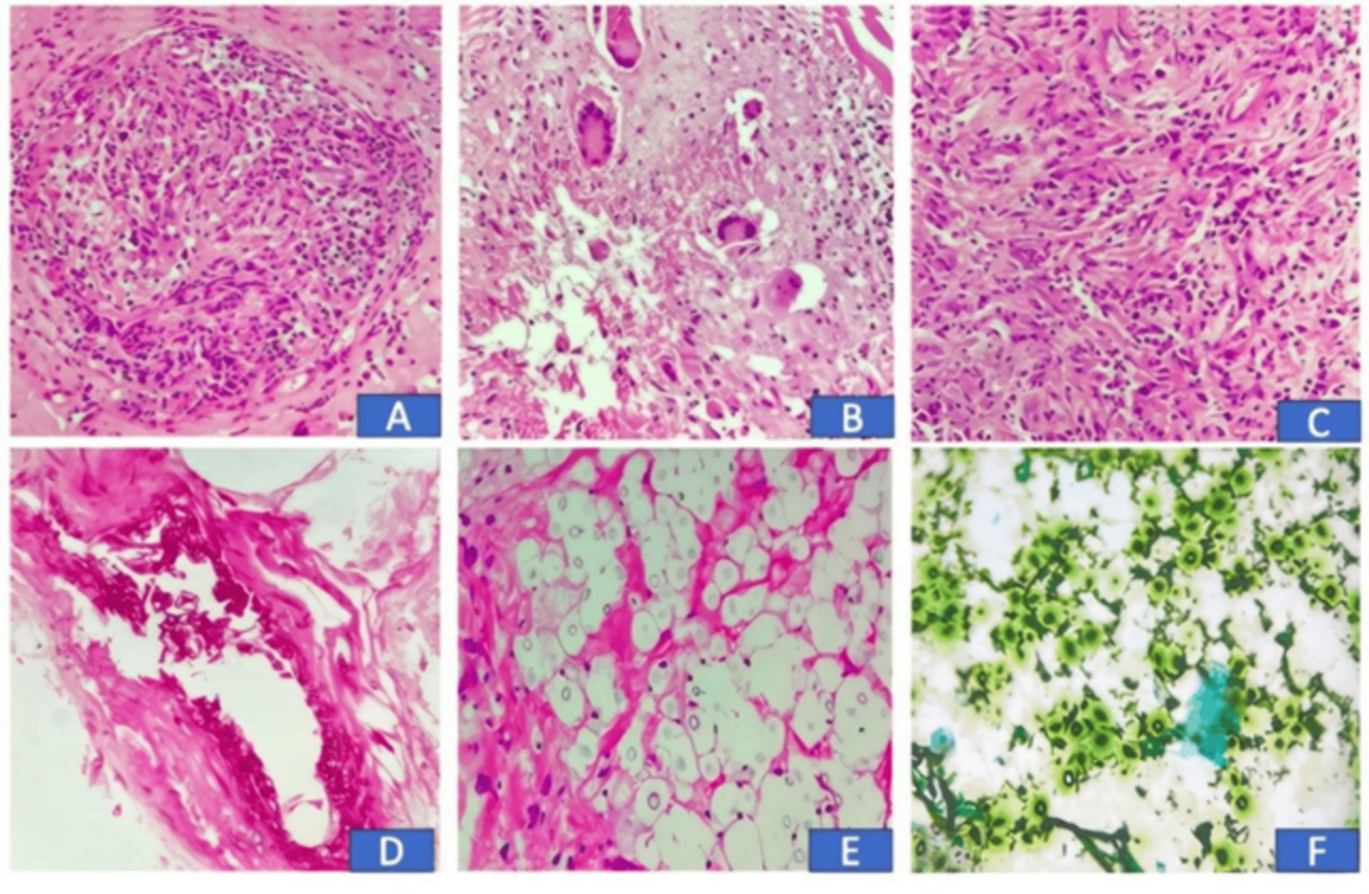
FIGURE 2: Haematoxylin and eosin (H&E) sections. (A) 40×, granuloma; (B) 40×, giant cells in a tuberculous abscess (Case 4); (C) 40×, inflammation in neuromelioidosis (Case 8); (D) 40×, vascular invasion by mucormycosis (Case 10); (E) 40×, yeast forms of Cryptococcus (Case 7); (F) 40×, GMS stain confirming Cryptococcus organisms (Case 7).

A broad spectrum of inflammatory and infectious lesions was observed on histopathological examination. A summary of individual case findings is presented in the master clinicopathological table (Table 6; Figure 2). Histopathological diagnoses are summarised in Table 5. Granulomatous inflammation without necrosis was identified in 3/12 cases (25.0%): Case 2, Case 5, and Case 11. Necrotising granulomatous inflammation consistent with tuberculosis was observed in 2/12 cases (16.7%): Case 4 and Case 9. Xanthogranulomatous inflammation was noted in 2/12 cases (16.7%): Case 1 and Case 3; CD68 immunopositivity was confirmed in Case 3. Chronic inflammatory process was seen in 2/12 cases (16.7%): Case 1 and Case 5. Organised bacterial abscess formation was observed in Case 6 (1/12, 8.3%). Acute-on-chronic inflammation consistent with melioidosis (*Burkholderia pseudomallei*) was seen in Case 8 (1/12, 8.3%). Cryptococcal infection was diagnosed in Case 7 (1/12, 8.3%). Mucormycosis caused by zygomycetes was identified in Case 10 and Case 12 (2/12, 16.7%). 

**Table 6:** Histopathological diagnoses in study patients (N = 12). Data are presented as N (%). Frequencies were compared using the chi-square (χ²) test where applicable; results are reported as χ² (df), p-value. Statistical significance threshold: p < 0.05. HPE: histopathological examination, df: degrees of freedom.

HPE Diagnosis	N	%	Cases
Granulomatous inflammation	3	25.0	2,5,11
Necrotising granulomatous inflammation (TB)	2	16.7	4,9
Xanthogranulomatous inflammation	2	16.7	1,3
Chronic inflammatory process	2	16.7	1,5
Acute on chronic inflammation (Melioidosis)	1	8.3	8
Organised bacterial abscess	1	8.3	6
Cryptococcal infection	1	8.3	7
Mucormycosis/fungal abscess (Zygomycetes)	2	16.7	10,12

Clinicopathological concordance

Clinicopathological concordance was observed in 9/12 cases (75.0%), as shown in Table 7. In 3/12 cases (25.0%), discordance was noted between the initial clinical and radiological impressions and the final HPE diagnosis. Chi-square analysis of the association between radiological impression (infective vs neoplastic vs indeterminate) and HPE category (infectious vs neoplasm) yielded χ² = 9.6, df = 2, p < 0.05, indicating a statistically significant association. In Case 7 and Case 11, radiological findings suggested metastatic adenocarcinoma and glioblastoma, respectively; however, HPE confirmed cryptococcal infection and tuberculosis, respectively. In Case 3, imaging was normal, but HPE revealed xanthogranulomatous inflammation. Despite negative microbiological studies, HPE confirmed mucormycosis in Case 10 and Case 12 through the identification of broad, aseptate fungal hyphae in tissue sections. 

**Table 7:** Clinicopathological concordance and discordance (N = 12). Data are presented as N (%). Association between radiological impression and HPE diagnosis was assessed using the chi-square (χ²) test: χ² = 9.6, df = 2, p < 0.05. Statistical significance threshold: p < 0.05. HPE: histopathological examination, df: degrees of freedom.

Concordance	N	%	Cases
Clinicopathological concordance	9	75.0	1,2,4,5,6,8,9,10,12
Clinicopathological discordance	3	25.0	3,7,11

## Discussion

Due to their similar clinical presentations, radiological characteristics, and inconsistent microbiological results, infections of the CNS and spinal cord pose a diagnostic challenge. The diversity of these infections is highlighted in this study, which recognises the crucial role of histopathological diagnosis in establishing an accurate diagnosis.

Granulomatous inflammation associated with or without necrosis accounted for five out of 12 patients (41.7%) and was the most common histological feature found in this study. Positive microbiological results, such as the GeneXpert assay and AFB staining, corroborated the necrotising granulomatous inflammation, which was highly suggestive of tuberculosis [11]. In a developing country like India, tuberculosis is still one of the most frequent causes of CNS infections in both immunocompetent and immunosuppressed populations [1,12]. Symptoms such as pain, back discomfort, vertebral degeneration, and neurological impairments are prominent clinical features of spinal tuberculosis [13]. Initiating definitive antitubercular therapy frequently requires histological or microbiological confirmation [8].

Xanthogranulomatous inflammation is an unusual inflammatory lesion with persistent inflammatory infiltrates and the presence of sheets of lipid-laden macrophages [14]. The lesion's histiocytic nature can be verified by CD68 immunohistochemistry. A significant percentage of patients in this series (25%) had fungal infections, indicative of the rising prevalence of opportunistic fungal infections in immunocompromised people [15]. The significance of early tissue detection in such individuals has been further highlighted by the rising incidence of mucormycosis in India, especially after the COVID-19 pandemic [16,17].

Radiological findings in one case suggested metastatic cancer, but the final diagnosis was cryptococcal infection, highlighting the need for prompt diagnosis and treatment [18]. Fungal stains are still necessary for precise identification because cryptococcal infections can radiologically resemble neoplastic tumours, particularly in immunocompromised hosts [4,7].

Infections due to Burkholderia pseudomallei are very rare. Neuromelioidosis is becoming more common in tropical areas such as India [19,20]. In this instance, the histopathological results were non-specific, demonstrating acute-on-chronic inflammatory changes; however, microbiological culture was used to make a conclusive diagnosis [5]. This instance emphasises how crucial it is to correlate microbiological and histological findings in order to make an appropriate diagnosis.

Non-specific chronic inflammatory lesions and organised bacterial abscesses further illustrate the wide range of pyogenic and chronic inflammatory CNS lesions seen in everyday practice [9,10,21].

Clinicopathological discordance in these cases needs to be analysed and interpreted based on sampling error, interpretation error, or the nature of the lesion. This will aid in understanding the biology of the disease. A high index of suspicion should be maintained to diagnose these infections accurately and avoid misinterpretation. A multidisciplinary approach with comprehensive clinical, laboratory, and histopathological evaluation is crucial to arrive at a timely diagnosis, as it has distinct treatment implications [1,11,20]. Limitations of the current study include the retrospective design and relatively small sample size, which constrain generalisability. Furthermore, comprehensive follow-up information or treatment outcomes were not available for all patients. To further assess the clinicopathological spectrum of CNS and spinal infections and reinforce the relevance of histopathological diagnosis in guiding treatment approaches, larger prospective multicentre studies are required. Molecular testing for the diagnosis of CNS infections using modalities such as multiplex PCR and metagenomic sequencing is also rapidly evolving in resource-rich settings. However, cost-effectiveness data and the involvement of policymakers are needed to identify how best these platforms can be used to improve the diagnosis of CNS infections in resource-limited settings [22].

Limitation

The present study has several limitations. First, the retrospective design and small sample size (n = 12) limit the generalisability and statistical power of the findings. Second, not all patients had comprehensive follow-up data or treatment outcome records available. Third, molecular diagnostic modalities such as multiplex PCR and metagenomic sequencing, which are rapidly evolving in better-resourced settings, were not uniformly applied; cost-effectiveness data and policy guidance are needed to determine how such platforms can best be integrated into resource-limited settings [22]. Larger, prospective, multicentre investigations are required to further define the clinicopathological spectrum of CNS and spinal infections and to validate the role of HPE-guided diagnosis.

## Conclusions

Our results highlight the value of a multidisciplinary strategy that incorporates microbiological investigations, neuroimaging, clinical findings, and thorough histological evaluation. The routine use of specific stains and immunohistochemistry in combination with routine haematoxylin and eosin staining substantially improved diagnostic accuracy, notably in cases of fungal and granulomatous lesions.

The diversity of neuroinfections poses enormous diagnostic challenges. A high index of suspicion must be maintained in the evaluation of infectious diseases of the CNS, even in immunocompetent individuals. Considerable overlap in clinicoradiological findings makes specific diagnosis difficult. Early diagnosis with appropriate imaging, laboratory confirmation, initiation of appropriate therapy, and multidisciplinary team discussion is warranted to prevent the morbidity and mortality associated with these diseases.

Early and accurate tissue diagnosis remains very important in the management of CNS and spinal infections because it allows timely and optimal treatment, which can eventually lead to better patient outcomes. Molecular diagnosis of CNS infections is still finding its place in routine clinical practice because it requires specialised laboratory facilities that are not yet widely available across Indian healthcare settings.
